# Visualisation of aortic flow disturbance in Marfan syndrome by 4D phase-contrast CMR

**DOI:** 10.1186/1532-429X-13-S1-P201

**Published:** 2011-02-02

**Authors:** Alex Pitcher, Tom E Cassar, Joseph Suttie, Jane M Francis, Paul Leeson, Edward Blair, B Paul Wordsworth, J Colin Forfar, Saul G Myerson, Michael Markl, Stefan Neubauer, Steffen E Petersen

**Affiliations:** 1 Oxford University, Oxford, UK

## Objective

To characterise the distribution, extent and severity of aortic flow disturbance in the aorta in Marfan syndrome.

## Background

Marfan syndrome commonly leads to progressive aortic dilation and aortic dissection, particularly at the aortic sinuses and descending thoracic aorta. Abnormal blood flow patterns may contribute to the enlargement and dissection of an inherently weak aorta.

## Methods

15 patients with Marfan syndrome and no history of prior aortic dissection or surgery, and 18 healthy volunteer controls matched for age, sex and height underwent CMR at 3T (Siemens, Erlangen). Time-resolved, 3D velocity-encoded and magnitude data were acquired using a phase contrast CMR sequence (Figure 2). Each dataset was evaluated for flow disturbance by two independent observers, experienced in aortic flow visualisation, and blinded to patient identity.

**Figure 1:**
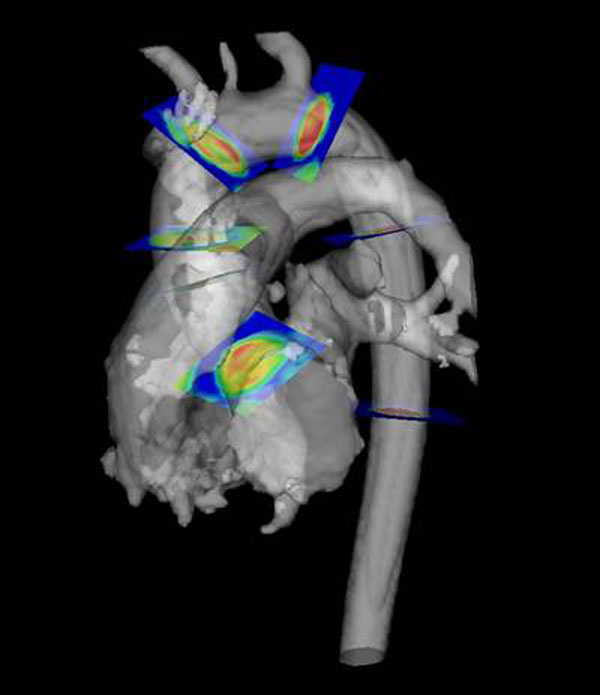
3D Phase-contrast image, with clip-planes dividing the aorta into six anatomical segments.

**Figure 2:**
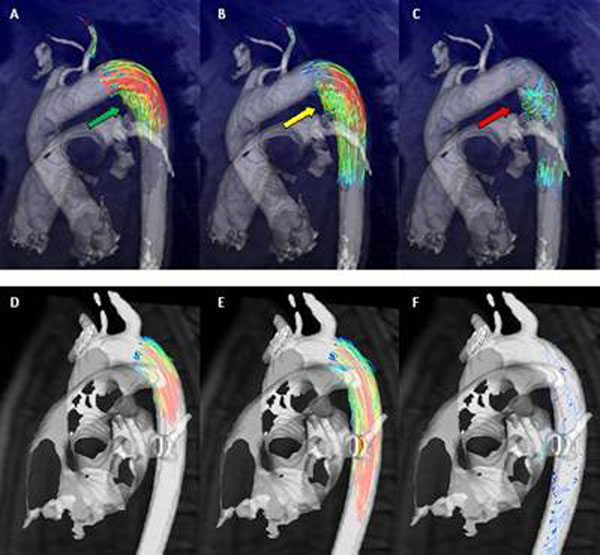
Still frames from a movie showing 4D flow images of the aorta of a patient with Marfan syndrome (top row, A-C), and a healthy volunteer (bottom row, D-F). Arrows highlight turbulent, complex vortex formation.

## Results

**Figure 3:**
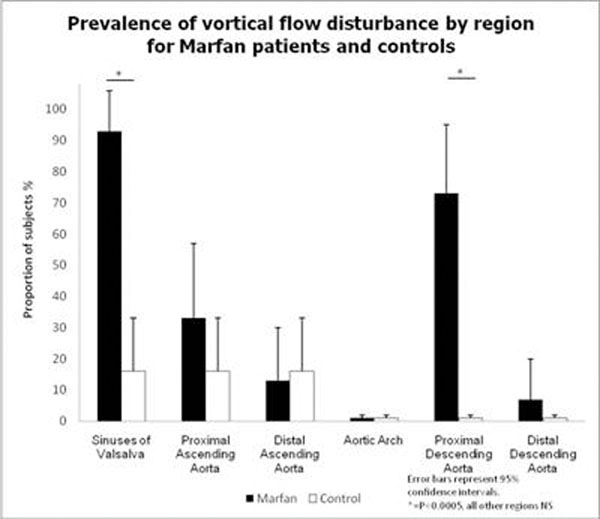
Prevalence of vortical flow disturbance by region for Marfan patients and controls.

Significant vortical flow in any segment (defined as flow disturbance occupying more than one half of the aortic lumen) was present in all patients with Marfan syndrome but in only 7/18 controls (P<0.0005). Severity of flow disturbance was greater in Marfan patients than controls (median severity score 3 for Marfan patients compared to 1 for controls, P<0.0005). There was marked regional variation in the prevalence of major flow disturbance (Figure 3), with the sinuses of Valsalva and proximal descending aorta being most frequently affected. Marfan subjects with more extensive flow disturbance tended to have larger and more angulated aortic diameters at that segment, but flow abnormalities were still frequent in those with only modestly enlarged segments.

We propose a classification of aortic flow disturbance in Marfan syndrome. Type A: flow disturbance confined to the sinuses of Valsalva, Type B: flow disturbance confined to the proximal descending aorta, Type C: flow disturbance in both the sinuses of Valsalva and the proximal descending aorta.

## Conclusion

Patients with Marfan syndrome commonly show aortic flow disturbance. The sinuses of Valsalva and proximal descending aorta are most frequently affected and these may contribute to aortic dilation and dissection seen at these regions in these patients.

